# The Gut Nexus: Unraveling Microbiota-Mediated Links Between Type 2 Diabetes and Colorectal Cancer

**DOI:** 10.3390/nu17233803

**Published:** 2025-12-04

**Authors:** Anns Mahboob, Chehbin Shin, Shahd Almughanni, Lubica Hornakova, Peter Kubatka, Dietrich Büsselberg

**Affiliations:** 1 Weill Cornell Medicine-Qatar, Doha P.O. Box 24144, Qatar; anm4019@qatar-med.cornell.edu (A.M.);; 2 Center of Experimental and Clinical Regenerative Medicine, Small Animal Clinic, University of Veterinary Medicine and Pharmacy, 041 81 Košice, Slovakia

**Keywords:** gut microbiota, type 2 diabetes mellitus, colorectal cancer, dysbiosis, short-chain fatty acids, bile acids

## Abstract

**Background/Objectives**: Colorectal cancer (CRC) and type 2 diabetes mellitus (T2DM) are two of the most rapidly rising chronic diseases globally. Despite appearing distinct, an emerging body of literature identifies shared etiopathogenic mechanisms mediated by gut microbiota. This review synthesizes 38 peer-reviewed studies to evaluate the compositional, metabolic, immune, and translational intersections of gut dysbiosis in the pathogenesis of T2DM-associated CRC. **Methods**: This narrative literature review examined 38 primary research articles (human and animal studies) retrieved from PubMed, Scopus, and Embase. Studies were selected based on relevance to the microbiota-mediated mechanisms linking T2DM and CRC, with a focus on compositional analysis, metabolomic shifts, immune activation, and therapeutic interventions. **Results**: The findings highlight a mechanistically rich overlap between T2DM and CRC through shared dysbiosis, characterized by depletion of SCFA-producing taxa (e.g., *Faecalibacterium*, *Roseburia*, *Butyricicoccus*), enrichment of pathobionts (e.g., *Fusobacterium nucleatum*, *Peptostreptococcus*), and the disruption of mucosal immunity and epithelial integrity. Metabolic shifts include reduced butyrate and increased toxic bile acids (e.g., deoxycholic acid), TMAO, and oxidative metabolites, while immune dysregulation features elevated LPS, IL-1β, CXCL3, and NF-κB signaling. Therapeutically, microbiota modulation via diet, metformin, and probiotics shows promise. **Conclusions**: Gut microbiota lies at the nexus of T2DM and CRC, functioning as a modifiable mediator rather than a passive bystander. Future research should prioritize longitudinal, multi-omic, and intervention-driven studies to enable personalized prevention and treatment strategies.

## 1. Introduction

Throughout the past 20 years, the role of gut microbiota and its impact on human health has gained increasing recognition [1]. A correlation between gut microbiota composition and the pathogenesis of a broad spectrum of diseases, ranging from metabolic disorders to neurodegenerative conditions, has been firmly established [2]. Delving even deeper, the mechanisms by which these diseases arise have also been elucidated at a molecular level [3]. What was once thought to primarily affect nutrition and lifestyle is now understood to influence the immune system and metabolic pathways as well.

Amongst the multitude of diseases affected by gut microbiota, this research focuses on two major public health challenges: colorectal cancer (CRC) and type 2 diabetes mellitus (T2DM). Recent meta-analyses confirm that specific shifts in taxa, such as the enrichment of *Escherichia-Shigella* and depletion of *Faecalibacterium prausnitzii*, are consistently associated with T2DM, underscoring the link between dysbiosis and disease [4]. Emerging evidence further implicates microbial metabolites, such as trimethylamine-N-oxide, secondary bile acids, hydrogen sulfide, and N-nitroso compounds, in CRC oncogenesis through DNA damage, inflammatory signaling, and immune modulation [3]. With rising global prevalence and significant morbidity and mortality, T2DM affects over 537 million people worldwide, projected to increase to 643 million by 2030 [5]. CRC also remains the third most common cancer globally and the second leading cause of cancer-related death [6]. Accumulating epidemiological evidence indicates that individuals with T2DM have a 20–30% higher risk of developing CRC [7]. Although shared lifestyle risk factors such as diet and obesity play a role, the gut microbiota has gained attention as a key mechanistic link between the two conditions in recent research.

Notably, the global landscape of CRC is shifting with an alarming rise in early-onset colorectal cancer (EOCRC), defined as diagnosis before age 50. EOCRC accounts for approximately 10% of all CRC cases and shows a particularly steep increase in developed countries [8]. While the causes remain multifactorial, growing evidence implicates gut dysbiosis and metabolic dysfunction—hallmarks of T2DM—as potential contributors to this trend [9]. The shared pathways of microbial dysbiosis, inflammation, and altered metabolite production suggest that T2DM may not only increase CRC risk overall but may also accelerate carcinogenesis in younger individuals. This highlights the urgency of understanding microbiota-mediated links to inform earlier screening and preventive strategies for at-risk populations.

The gut microbiota comprises trillions of microorganisms, influencing host metabolism, immune regulation, and epithelial integrity [10]. In T2DM, dysbiosis contributes to insulin resistance, systemic inflammation, and altered gut permeability. In CRC, it contributes to tumor initiation, immune evasion, and chemoresistance [11]. The overlap in microbial disruptions raises the possibility of a common mechanistic axis driving both diseases, potentially amplifying the threat of EOCRC in diabetic populations. Of particular interest are butyrate-producing genera such as *Roseburia* and *Coprococcus*: their depletion impairs colonocyte energy homeostasis and anti-inflammatory defenses, while butyrate itself acts as a histone deacetylase inhibitor with direct antiproliferative effects in CRC cells [12,13,14,15,16].

Nevertheless, the diversity of the gut microbial community and the complexity of defining “dysbiosis” complicate efforts to pinpoint shared mechanisms. Moreover, newly proposed therapeutic frameworks, like the metformin-gut microbiota-CRC axis, highlight how metformin’s beneficial effects on CRC risk may be microbiota-mediated, adding a potential pharmaco-microbial intervention angle [17]. This review explores specific gut microbial taxa and downstream processes—including metabolic byproducts, immune responses, and inflammatory markers—implicated in T2DM and CRC. By synthesizing findings from human and animal studies, we aim to outline converging pathways, identify knowledge gaps, and highlight therapeutic strategies such as microbiota modulation, with implications for mitigating CRC risk, particularly in younger diabetic cohorts.

## 2. Materials and Methods

### 2.1. Search Strategy

This narrative review was informed by a structured literature search. PubMed, Embase, and Scopus were searched for studies using combinations of the following terms: “type 2 diabetes”, “T2DM”, “colorectal cancer”, “CRC”, “gut microbiota”, “microbiome”, “short-chain fatty acids”, “bile acids”, “Fusobacterium”, “metformin”, “probiotics”, “prebiotics”, and “fecal microbiota transplantation”. Reference lists of retrieved articles were also screened.

### 2.2. Inclusion and Exclusion Criteria

We included peer-reviewed human or animal studies examining gut microbiota composition, microbial metabolites, or microbiota-targeted interventions in the context of T2DM, CRC, or both. We excluded reviews, conference abstracts, editorials, non-English articles, and studies lacking microbiota or metabolite measurements.

### 2.3. Study Selection and Data Extraction

A total of 38 studies met the inclusion criteria. For each, we extracted study design, population, microbiota platform, microbial/metabolite changes, CRC-related findings, and metabolic parameters. The extracted data are summarized in Table 1. Due to heterogeneity, no quantitative pooling was possible.

## 3. Results

### 3.1. Microbiota Composition Across T2DM, CRC, and T2DM+CRC

**Figure 1:**
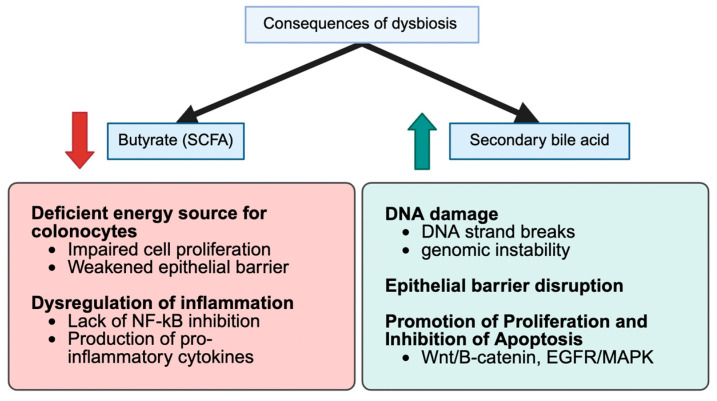
Conceptual Schematic of Convergent Disbiosis Across Healthy, T2DM, CRC, and DCRC States. This figure summarizes characteristic microbial and metabolite patterns across states, including depletion of SCFA-producing taxa (*Faecalibacterium*, *Roseburia*, *Butyricicoccus*) and enrichment of pro-inflammatory or oncogenic taxa (*Fusobacterium*, *Parvimonas*, *Peptostreptococcus*). SCFA reductions and increases in secondary bile acids (e.g., DCA) and TMAO are shown. This is a conceptual, literature-derived schematic; supporting data appear in Table 1, Table 2 and Table 3 and Section 3.2 and Section 4.2.

Mounting evidence shows that both type 2 diabetes mellitus (T2DM) and colorectal cancer (CRC) are associated with special patterns of gut microbial imbalance, with some similarities. In T2DM, beneficial butyrate-producing Clostridiales like *Faecalibacterium prausnitzii*, *Roseburia*, and *Eubacterium rectale* are consistently reduced. Likewise, the mucin-degrading *Akkermansia muciniphila* is depleted. Instead, harmful bacteria, such as Proteobacteria, Enterobacteriaceae, and particular species, become dominant [11,32,33,34,35]. CRC, on the other hand, brings its microbial fingerprint. Pro-carcinogenic species, such as Fusobacterium nucleatum, Porphyromonas, Peptostreptococcus, and Parvimonas, are enriched, while protective commensals are reduced [35,36,37,38,39]. In individuals with both DCRC, these dysbiotic features are more pronounced and distinct. These patients tend to have more *Eggerthella*, *Hungatella*, and *Parvimonas*, and fewer *Butyricicoccus*, *Lactobacillus* spp., and *Paraprevotella* [40,41,42,43]. These microbial patterns have been reported in human studies and explored in experimental models, although translational and causal implications remain under investigation [43,44,45]. Interestingly, interventions such as Gegen Qinlian decoction have shown promise in experimental diabetic CRC mouse models by improving microbial balance and correlating with reduced tumor burden, but their applicability to human disease remains to be clarified, particularly in microsatellite-stable cases [34] (Figure 1).

**Table 2:** Recurrent microbial taxa and metabolites altered across type 2 diabetes mellitus (T2DM), colorectal cancer (CRC), and combined DCRC contexts in the included literature.

Taxon/Metabolite	Qualitative Direction Across T2DM/CRC/DCRC	Representative Studies
*Faecalibacterium* spp.	Generally ↓ in CRC; often reduced in metabolic disorders	[22]
*Roseburia* spp.	↓ in CRC and obesogenic states; SCFA-producing	[22]
*Butyricicoccus* spp.	↓ in DCRC vs. controls	[18]
*Lactobacillus* spp.	Decreased in some DCRC cohorts; context-dependent	[18]
*Paraprevotella* spp.	↓ in DCRC	[18]
*Eggerthella* spp.	↑ in DCRC; enriched in dysbiosis	[18]
*Hungatella* spp.	↑ in DCRC	[18]
*Peptostreptococcus* spp.	↑ in DCRC; associated with CRC and inflammation	[18]
*Parvimonas* spp.	↑ in DCRC and CRC	[18]
*Veillonella* spp.	↑ in DCRC; associated with chemoresistance in CRC	[18]
*Akkermansia muciniphila*	↓ in obesity/T2DM; often ↑ after beneficial interventions	[27,28,31]
SCFAs (total)	Often ↓ in CRC and dysmetabolic states; ↑ with fiber interventions	[20,24,30]
Butyrate	↓ in CRC/dysbiosis; ↑ with high-fiber/prebiotic intake	[20,21,22]
Acetate	Altered in DCRC; direction varies by study	[18,20]
Propionate	Altered SCFA profile; direction varies	[29,30]
Total bile acids	Dysregulated in DCRC and T2DM–CRC	[18,26]
Secondary bile acids (e.g., DCA)	↑ in DCRC/CRC; tumor-promoting	[18,26,30]
TMAO	↑ or altered in DCRC; pro-atherogenic and potentially pro-tumorigenic	[18]
ROS and oxidative stress markers	↑ in dysbiosis and SCFA/BA imbalance contexts	[20,46]
Inflammatory cytokines (TNF-α, IL-6, IL-1β, etc.)	↑ in obesity, T2DM, and dysbiotic CRC	[27,30]

↓, ↑ = Decrease, Increase, respectively.

**Table 3:** Dietary and non-dietary strategies reported to modulate gut microbiota composition and colorectal cancer (CRC)-related outcomes, with relevance to type 2 diabetes mellitus (T2DM) and metabolic states.

Intervention/Strategy	Type	Context	Microbiota Effects	CRC-Related Outcomes	Metabolic/T2DM-Related Outcomes	Therapeutic Implications	Reference
Garlic intake	Dietary	Human; observational	Alters blood/gut microbiome signatures; supports beneficial taxa and anti-inflammatory profile	Medium/high garlic intake linked to reduced CRC risk	Associated with better inflammatory and metabolic parameters	Supports garlic-rich diets as microbiota- and immune-modulating chemopreventive strategy	[19]
Prebiotic fiber supplementation	Dietary	Human; prospective cohort	Expected enrichment of *Bifidobacterium* and SCFA-producing taxa	Associated with modestly lower CRC risk and improved post-CRC survival	Linked to better cardiometabolic profiles	Supports high-fiber/prebiotic intake as part of CRC and metabolic risk reduction	[21]
High-fiber, plant-rich diet in obese patients with prior adenomatous polyps	Dietary	Human; interventional	Increases SCFA producers; reduces pro-inflammatory/pathogenic taxa	Reduces markers and intermediate endpoints related to colon cancer risk	Improves weight, insulin sensitivity, and lipid profile	Fiber-rich diets provide dual protection for colon and metabolic health in high-risk populations	[24]
High-fat Western-style diet	Dietary	Mouse; HFD models	Reduces diversity, enriches pro-inflammatory taxa, disrupts barrier	Favors a pro-tumorigenic colonic environment and may increase CRC susceptibility	Promotes insulin resistance, obesity, and T2DM-like metabolic dysfunction	Reducing HFD and replacing with fiber-rich patterns may lower both CRC and T2DM risk	[25,46]
SCFA-enhancing strategies (high fiber, prebiotics)	Dietary/microbiota-directed	In vitro, human, and animal data	Increase butyrate and other SCFAs; enrich butyrate-producing taxa	SCFAs inhibit CRC cell proliferation and induce apoptosis; improve barrier	SCFAs improve insulin sensitivity and energy homeostasis	Reinforces dietary/prebiotic strategies to increase SCFA production as a shared T2DM–CRC target	[20,30]
*Akkermansia muciniphila* supplementation/EVs	Microbiota-directed	Mouse and preclinical	Increases *A. muciniphila*, improves mucus layer and tight junctions; modulates TLR signaling	Not yet directly tested in CRC models, but reduces inflammatory milieu that favors carcinogenesis	Reduces obesity-related inflammation and improves insulin sensitivity	Promising candidate for next-generation probiotics aimed at obesity, T2DM, and CRC prevention	[27,28,31]
Metformin therapy	Pharmacologic	Mouse tumor models; human epidemiology (cited in text)	Modulates gut microbiota, increasing some beneficial taxa and altering SCFA/BA profiles	Reduces tumor growth in preclinical models; observational data suggest lower CRC risk in metformin users	Improves glycemic control and insulin resistance in T2DM	Supports potential dual benefit of metformin on metabolic and oncologic outcomes, though causal links remain to be confirmed	[29]
General high-fiber/Mediterranean-style diets	Dietary	Human/review	Enrich SCFA-producing taxa, increase microbial diversity, reduce bile acid dysregulation	Associated with lower CRC risk in epidemiologic studies	Linked to lower risk of obesity, insulin resistance, and T2DM	Support guidelines promoting plant-rich, high-fiber patterns to jointly address CRC and T2DM burdens	[30]

### 3.2. Metabolic Pathways & Microbial Metabolites

Gut bacteria produce numerous metabolites that directly impact the host’s metabolism and risk of CRC. One of the most critical groups, short-chain fatty acids (SCFAs), especially butyrate, are often found in small concentrations in individuals with T2DM or CRC. Butyrate helps maintain gut barrier integrity and reduces inflammation, and its loss contributes to disease progression [12,32,34,47]. Meanwhile, diabetogenic microbiota affect bile acid metabolism, converting more primary bile acids into harmful secondary bile acids, such as deoxycholic acid. DCA is a compound known to damage DNA and promote tumor growth [18,48]. Additionally, levels of trimethylamine-N-oxide (TMAO), a gut-derived metabolite of dietary choline and carnitine, are elevated in individuals with diabetes and are linked to oxidative stress and the proliferation of colon cells [18]. Other metabolic shifts include increased amino acid breakdown and the production of reactive oxygen species, which creates a greater risk to gut health [41,45] (Figure 1).

### 3.3. Immune and Inflammatory Modulation

Chronic inflammation connects T2DM and CRC. A disrupted microbiota impairs the gut barrier, allowing microbial products, such as lipopolysaccharide (LPS), to enter the circulation and provoke systemic inflammation [33,49]. This leads to elevated inflammatory markers, including IL-1β and CXCL3, as well as the overactivation of enzymes such as NOX4. Probiotics—particularly *Bifidobacterium* and *Lactobacillus* spp.—can help restore balance and reduce inflammatory cytokines in patients with diabetes [33,38,50,51]. In CRC, bacteria such as *F. nucleatum* trigger TLR/NF-κB signaling and inhibit immune cell responses that are responsible for tumor growth suppression [35,52]. Dietary elements, such as garlic, and certain medications, including metformin, not only modulate the gut microbiome but also reduce oxidative stress and inflammation in CRC models [33,39,53] (Figure 1).

### 3.4. Metabolic Dysfunction Amplified by Dysbiosis

Gut dysbiosis can worsen the metabolic disturbances already present in diabetes. High blood sugar and insulin levels activate growth-promoting pathways, such as PI3K/Akt/mTOR, in colon cells, which increases the risk of cancer [4,47]. Microbial LPS also stimulates TLR4, worsening insulin resistance [8,54]. Moreover, experimental data show that transferring diabetic gut microbiota into healthy mice increases their risk of developing colon tumors [38,55]. These findings reinforce the idea that dysbiosis is not just a byproduct but also acts as a driving force in metabolic and cancer pathology (Figure 1).

### 3.5. Interventions Targeting Microbiota

Multiple methods have shown promising effects in rebalancing the microbiota and reducing CRC risk in diabetic individuals. High-fiber diets increase the production of SCFA-producing bacteria and GLP-1 secretions, thereby improving insulin sensitivity and colon health [41,56]. Prebiotics, such as garlic, similarly promote a healthier gut environment and protect against tumor growth [39]. Drugs such as metformin not only lower blood glucose but also promote the development of beneficial bacteria, including Akkermansia, which reinforces the gut barrier and reduces cancer risk [13,57]. Observational studies have also shown that metformin users have lower rates of CRC [13]. Additionally, probiotics such as Lactobacillus casei and Akkermansia muciniphila enhance the mucosal barrier and reduce inflammation [58,59]. Experimental therapies, including fecal microbiota transplantation (FMT), are currently under investigation and are recognized as additional methods that could restore a balanced gut microbiota [60] (Figure 1).

### 3.6. Diagnostic and Research Applications

Current innovations facilitate earlier detection and improved risk prediction of CRC in metabolic disease contexts. The SNIG method and mbImpute algorithm both enhance the ability to identify early shifts in gut microbiota that precede the onset of CRC [61,62]. Studies have also revealed regional disparities in microbiota and CRC outcomes—for instance, in Appalachia versus other U.S. populations, highlighting the need for localized, personalized approaches [51,63]. Global research efforts continue to grow, focusing on metagenomics, dietary modifications, and microbiota-informed screening [64] (Figure 1).

## 4. Discussion

### 4.1. Microbiota as a Convergent Axis in T2DM and CRC

This review underscores that gut microbiota dysbiosis represents a convergent mechanistic axis linking type 2 diabetes mellitus (T2DM) with colorectal cancer (CRC). Across 38 studies, we found overlapping microbial alterations in both conditions, characterized by loss of beneficial commensals and enrichment of pro-pathogenic taxa. In both T2DM and CRC, health-promoting short-chain fatty acid (SCFA) producers such as *Faecalibacterium prausnitzii*, *Roseburia*, and *Eubacterium rectale* are consistently depleted [8]. These bacteria typically support intestinal barrier function and modulate inflammation, so their reduction is a common marker of dysbiosis in metabolic and neoplastic disease. Because most data included in this review are observational, cross-sectional, or preclinical, the associations described herein should not be interpreted as definitive causality; instead, they highlight promising biological and mechanistic hypotheses requiring validation through longitudinal and interventional trials.

Conversely, both T2DM and CRC exhibit overgrowth of opportunistic or inflammatory genera. For instance, CRC-associated microbes, such as Fusobacterium nucleatum, Peptostreptococcus (including *P. anaerobius*), Porphyromonas, and Parvimonas, are frequently detected at elevated levels in tumor patients [65]. T2DM guts tend to harbor increases in endotoxin-producing *Proteobacteria* and certain *Enterobacteriaceae*, which similarly drive inflammation. Notably, in patients suffering from both T2DM and CRC, gut microbiome profiles are not merely an additive combination but rather a synergistic derangement. A recent metagenomic study revealed that patients with CRC and T2DM harbor a unique microbiota signature, characterized by higher abundances of Eggerthella, Hungatella, Peptostreptococcus, and Parvimonas, alongside a marked depletion of SCFA-producing genera, including *Butyricicoccus*, *Lactobacillus* spp., and *Paraprevotella* [34]. These compounded shifts indicate that T2DM is associated with a gut microbial profile that overlaps substantially with CRC-linked dysbiosis, potentially creating conditions favorable for pathophysiological convergence.

Reduced diversity is also observed; CRC patients generally have a less diverse microbiota than healthy controls, and T2DM can further skew this diversity through dietary and metabolic influences [66]. Altogether, the convergent dysbiosis seen in T2DM and CRC reflects shared microbial patterns that may contribute to overlapping metabolic disturbances and CRC-relevant pathways. By reflecting and exacerbating host pathophysiology, the microbiota changes in these conditions strengthen the biological link between them (Figure 1).

### 4.2. Metabolomic Shifts and Carcinogenic Potential

Microbiota-derived metabolites have been proposed as possible mechanistic links in the association between T2DM and CRC. A salient example is the consistent reduction in SCFAs (especially butyrate) in both diabetic and CRC cohorts [18]. Butyrate, produced by fiber-fermenting gut bacteria, serves as an energy source for colonocytes and exerts anti-tumor effects by reinforcing epithelial barrier integrity and dampening inflammation [67]. In both diseases, butyrate scarcity can compromise mucosal health and immune tolerance, potentially accelerating the progression of neoplasms.

Meanwhile, dysbiotic microbiota in T2DM and obesity favor altered bile acid metabolism. High-fat diets and diabetic microbiomes increase the conversion of primary bile acids into carcinogenic secondary bile acids, such as deoxycholic acid (DCA) and lithocholic acid [11]. DCA is a known genotoxic agent that damages DNA in colonic cells and promotes tumorigenesis by inducing oxidative stress and epithelial proliferation [11]. In diabetic CRC patients, metabolomic analyses confirm elevated fecal DCA and 12-keto-lithocholic acid levels, alongside the SCFA deficiencies [18].

Another metabolite of interest is trimethylamine-N-oxide (TMAO), a byproduct of gut microbial metabolism of choline- and carnitine-rich foods [45]. TMAO levels are often elevated in T2DM and have been “strongly linked” to colorectal cancer risk in humans [68]. Mechanistically, TMAO promotes a pro-carcinogenic environment by activating inflammatory pathways (e.g., NF-κB) and inducing oxidative stress and NLRP3 inflammasome activation [45]. Recent work has demonstrated that TMAO can directly enhance the proliferation and angiogenesis of colorectal cancer cells, highlighting its potential as an oncogenic factor [36].

Beyond these, diabetogenic microbiomes also yield excess branched-chain amino acids and endotoxins that contribute to insulin resistance and DNA damage. Even microbial genotoxins play a role: specific *Escherichia coli* in CRC carry the *pks* island, producing colibactin, a genotoxin causing DNA alkylation and mutations, a mechanism not classically tied to T2DM but emblematic of microbiota-driven mutagenesis in CRC [69].

Notably, the loss of key butyrate-producing genera such as *Roseburia*, *Faecalibacterium prausnitzii*, and *Coprococcus* is a recurrent finding in both T2DM and CRC cohorts [53,67,70]. Their depletion impairs colonocyte energy homeostasis, reduces epithelial barrier protection, and attenuates anti-inflammatory defenses. Butyrate itself also acts as a histone deacetylase (HDAC) inhibitor, exerting direct antiproliferative and pro-apoptotic effects in CRC cells [8,71]. Conversely, the overrepresentation of pathobionts, such as *Fusobacterium nucleatum* and *Eggerthella lenta*, is frequently observed in CRC and metabolic disorders, where they drive pro-inflammatory signaling and DNA damage [1].

**Figure 2:**
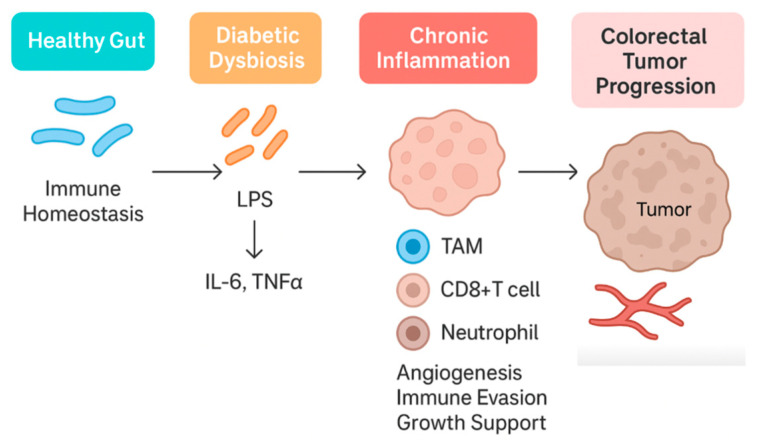
Proposed metabolic–oncogenic pathways linked to gut microbiota alterations. A conceptual model illustrating hyperinsulinemia, IGF-1 signalling, PI3K/Akt/mTOR activation, bile acid dysregulation, SCFA deficiency, and microbial-driven metabolic reprogramming. This schematic summarizes recurrent themes and is not derived from a single dataset.

In summary, the metabolomic shifts in dysbiosis—from loss of protective SCFAs to accumulation of bile acids, TMAO, and other toxins—create a biochemical milieu that favors colon tumor initiation and growth (Figure 2). Importantly, many of these metabolites (butyrate, DCA, and TMAO) also intersect with metabolic disease pathways, underscoring how one microbiota-driven biochemical imbalance can contribute to the development of both diabetes and cancer [72].

### 4.3. Immune Dysregulation and Barrier Breakdown

Chronic low-grade inflammation is a well-established link between metabolic syndrome and cancer, and the gut microbiome plays a significant role in this immune dysregulation [73]. Both T2DM and CRC are associated with a “leaky gut” phenomenon, wherein dysbiosis compromises the intestinal barrier, allowing microbial products, such as lipopolysaccharide (LPS), to translocate into the circulation. In metabolic disease, this microbial endotoxemia triggers systemic inflammation via Toll-like receptor 4 (TLR4) and NF-κB signaling, contributing to insulin resistance and tissue damage [8]. For instance, obese diabetic mice exhibit elevated serum LPS, which correlates with inflammation in both hepatic and adipose tissue, while treatments that modulate microbiota reduce LPS and restore barrier function (e.g., prebiotics enhancing tight-junction proteins) [74,75].

In the colon, LPS and other pathogen-associated molecules activate mucosal immune cells to release pro-inflammatory cytokines (e.g., IL-1β, IL-6, TNF-α), creating an environment conducive to tumor promotion [76]. Diabetic mice indeed show increased colonic IL-1β expression and neutrophil-recruiting chemokines (like CXCL3) in parallel with dysbiosis [73]. The same study demonstrated overactivation of the enzyme NADPH oxidase 4 (NOX4) in diabetic colon tissue, linking microbial imbalance to oxidative stress in the epithelium [73].

In CRC, an impaired immune surveillance is often observed in tumor microenvironments enriched with certain bacteria. *Fusobacterium nucleatum*, strongly associated with CRC progression, can drive a tumor-promoting immune profile through multiple mechanisms. It induces persistent local inflammation (via IL-1β, IL-6, and TNF-α upregulation) and concurrently suppresses anti-tumor immunity: *F. nucleatum* adhesins (e.g., FadA and Fap2 proteins) allow it to bind colonocytes and immune cells, disrupting E-cadherin junctions and engaging T cell inhibitory receptors (like TIGIT) [77]. The result is dampened cytotoxic T/NK cell activity and immune evasion by the tumor [78].

Thus, dysbiosis not only fuels inflammation but also skews immune responses toward tumor tolerance. In diabetic states, systemic inflammation and altered gut microbes may further impair anti-tumor immune vigilance, for example, by increasing the number of myeloid-derived suppressor cells or inflammatory macrophages in circulation. The combined effect is a breakdown of normal immune-mediated tumor suppression.

**Figure 3:**
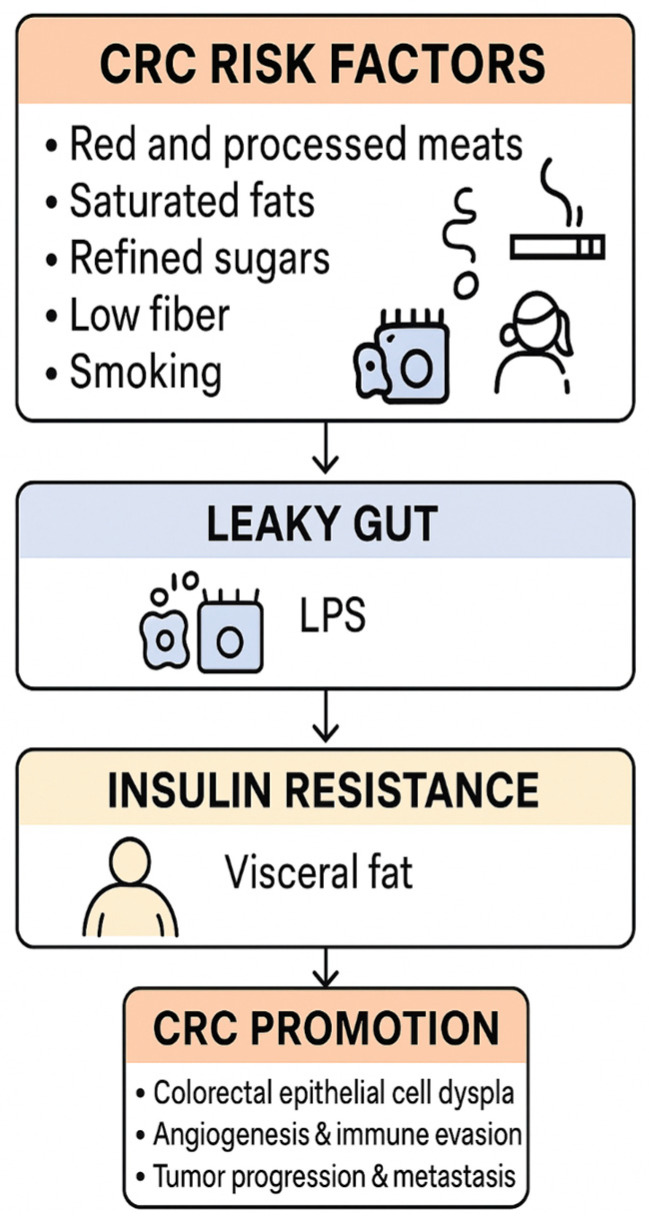
Inflammatory and Immune Pathways Linking Dysbiosis, T2DM, and CRC. Highlights LPS–TLR4 signalling, IL-6, TNF-α, IL-1β, CXCL3, NOX4, epithelial barrier compromise, and adipose-driven inflammation. Although visceral adiposity is established in CRC risk, specific data on pericolic fat remain limited. Conceptual schematic.

In essence, gut microbial dysbiosis seeds a pro-inflammatory, immunosuppressive loop: increased gut permeability and LPS drive metabolic inflammation. At the same time, CRC-associated bacteria exploit that inflamed milieu to blunt local immune attack (Figure 3). This immune crosstalk, in part, explains why T2DM confers worse CRC outcomes—the body’s defenses are subverted on multiple levels by the microbiota.

### 4.4. Obesity and Hyperinsulinemia: A Feed-Forward Loop

Obesity and insulin resistance, common in type 2 diabetes mellitus (T2DM), further amplify the microbiota–cancer axis in a vicious cycle. Adipose tissue in obesity releases adipokines (like IL-6, leptin, TNF-α) that sustain systemic inflammation and can alter gut microbiota composition. Conversely, gut dysbiosis in high-fat diet contexts increases energy harvest and promotes weight gain, reinforcing obesity. This bidirectional interaction creates a feed-forward loop of metabolic deterioration.

A central feature is hyperinsulinemia: T2DM patients often exhibit elevated insulin and IGF-1 levels, particularly in the early stages of the disease, which serves as a growth signal for epithelial cells [79]. Colonocytes exposed to excess insulin/IGF-1 activate the PI3K/Akt/mTOR pathway, accelerating cellular proliferation and inhibiting apoptosis [79]. Our findings align with prior reports that chronic hyperinsulinemia can directly drive colon tumorigenesis via these mitogenic pathways [47].

At the same time, dysbiosis exacerbates insulin resistance through mechanisms such as LPS-induced TLR4 activation in insulin-responsive tissues, driving NF-κB-mediated inflammation that impairs insulin signaling [8]. The result is a self-perpetuating circuit: microbial LPS and nutrient metabolites worsen metabolic control, leading to higher glucose and insulin levels, which in turn create a pro-neoplastic internal environment (fueling DNA synthesis, promoting advanced glycation end-products, etc.). Supporting a causal role, germ-free or antibiotic-treated mice colonized with the microbiota from diabetic donors develop both metabolic disturbances and a higher burden of preneoplastic colonic lesions compared to those receiving microbiota from healthy donors [80]. In one experiment, the transplantation of feces from T2DM mice into wild-type mice resulted in increased intestinal polyp formation, demonstrating that the diabetic microbiome can transmit a cancer-prone phenotype [80].

Obesity further exacerbates this effect by creating a chronic inflammatory environment (with elevated TNF-α and IL-1β from adipose tissue) that synergizes with microbiota-driven inflammation. Additionally, obese-diabetic states often involve gut microbiomes that produce more secondary bile acids (as discussed) and less GLP-1, factors that, respectively, promote carcinogenesis and worsen glycemic control [35]. Recent clinical studies also indicate that GLP-1 receptor agonists (e.g., semaglutide) not only improve glycemic control but can partially normalize dysbiotic signatures, reducing pro-inflammatory taxa and restoring butyrate producers [81]. This highlights a potential therapeutic avenue to disrupt the cycle.

All these elements intertwine into a dangerous positive feedback loop. In summary, the co-existence of obesity and T2DM creates a metabolic–microbial milieu that actively propels colon carcinogenesis: it is a loop wherein dysbiosis begets insulin resistance and inflammation, and those metabolic changes in turn foster further dysbiosis and unrestrained epithelial growth (Figure 2). Breaking this cycle is therefore critical in high-risk individuals. While visceral obesity is a well-established risk factor for CRC, specific data on ectopic pericolic fat as an immunometabolic niche that modulates CRC risk—particularly in individuals with T2DM—are scarce, and this remains an important but underexplored research question.

### 4.5. Therapeutic Modulation and Translational Insights

The recognition of gut microbiota as a linchpin in T2DM-related colorectal oncogenesis opens promising therapeutic avenues. Modulating the microbiome—through diet, drugs, or direct microbial interventions—can potentially tackle the twin challenges of metabolic control and cancer prevention. Dietary strategies are foundational. High-fiber diets, for example, enrich SCFA-producing bacteria and have been shown to raise colonic butyrate levels while improving glucose homeostasis [18]. Increasing the intake of fermentable fiber (through fruits, vegetables, and whole grains) in diabetic patients can restore populations of Faecalibacterium and Bifidobacterium, leading to enhanced production of GLP-1 and PYY, which improves insulin sensitivity and reduces colon inflammation [76]. Prebiotic supplementation offers a concentrated approach, specifically stimulating beneficial microbes with inulin-type fructans, resistant starches, or oligosaccharides. This approach has demonstrated reductions in systemic inflammatory markers and glycemic levels in T2DM, alongside anti-tumorigenic effects in murine CRC models (by increasing butyrate) [45]. Intriguingly, even specific foods like garlic have microbiome-mediated benefits. Garlic contains fructooligosaccharides and organosulfur compounds that can modulate gut flora—epidemiological data suggest high garlic consumption correlates with lower CRC risk, potentially via enriching protective microbes and inhibiting carcinogenic species [82]. Traditional Chinese herbal remedies are also being explored in this context. Gegen Qinlian Decoction (GQD), a botanical formula, has demonstrated the ability to remodel the gut microbiota and enhance the intestinal barrier. In diabetic CRC mouse models, GQD treatment increased mucin-degrading *Akkermansia muciniphila* and SCFA-producers, reduced *Fusobacterium* load, and was associated with reduced tumor numbers and sizes (particularly in microsatellite-stable CRC, which is often resistant to immunotherapy) [40]. One study even found that adding GQD to therapy enhanced the response to anti-PD-1 immunotherapy in MSS CRC by shifting the microbiome toward a more immunostimulatory profile [40]. Such findings underscore the potential of microbiota-directed adjuvants in oncology.

Beyond single nutrients, entire dietary patterns exert profound effects on the gut microbiota and are closely linked to CRC risk. Western-style diets, characterized by high intakes of red and processed meat, saturated fat, and refined carbohydrates, are associated with reduced microbial diversity, depletion of SCFA-producing taxa, and enrichment of bile acid-transforming and pro-inflammatory bacteria, changes that are repeatedly linked to colorectal carcinogenesis [PMID: 29333111, PMID: 35760086]. In contrast, Mediterranean-style and other plant-rich, high-fibre dietary patterns enrich genera such as *Faecalibacterium*, *Bifidobacterium*, and *Roseburia*, increase butyrate production, and are associated with lower CRC incidence in epidemiological studies [PMID: 29867803, PMID: 32359135]. In individuals with T2DM, adopting such fibre-rich patterns is therefore likely to confer dual benefit, improving glycaemic control and cardiometabolic status while attenuating microbiota-mediated CRC risk.

Metformin is a particularly interesting example of a drug that may interface with both metabolic and oncologic pathways. Epidemiological data suggest that metformin use in patients with T2DM is associated with lower CRC incidence compared with some other glucose-lowering regimens, although confounding by indication cannot be excluded [45,83]. Preclinical studies indicate that metformin can remodel gut microbiota composition, increase SCFA production, and modulate bile-acid and inflammatory signalling, in parallel with its well-known insulin-sensitising effects [57]. Nonetheless, most of the human evidence remains observational, and it is not yet possible to assign a definitive causal role to metformin-induced microbiota changes in mediating CRC risk reduction. Larger, mechanistically informed trials will be required to clarify whether microbiota modulation contributes meaningfully to metformin’s putative anticancer effects.

Probiotics offer another direct strategy. The administration of beneficial strains, such as Lactobacillus casei, Bifidobacterium longum, or Akkermansia, has been shown to attenuate metabolic endotoxemia and inflammation in diabetic models [83]. In rodent studies, probiotic treatment restored colon length and reduced polyp formation in diabetic mice by replenishing butyrate producers and lowering colonic IL-1β levels [45]. Clinically, probiotics and synbiotics (a combination of probiotics with prebiotic fibers) have shown promise in improving postoperative recovery in patients undergoing CRC surgery and in enhancing the efficacy of chemotherapy and immunotherapy [83]. For example, perioperative probiotic use has been associated with lower rates of surgical site infections and faster return of bowel function in colorectal surgery [45].

Looking forward, more experimental interventions are on the horizon. Fecal microbiota transplantation (FMT), the transfer of stool from a healthy donor, is being tested in clinical trials as an adjunctive therapy to improve insulin sensitivity in metabolic syndrome and increase responsiveness to cancer immunotherapies. Preliminary data suggest FMT can engraft new microbial communities that reduce inflammation and even modulate the tumor microenvironment favorably (for instance, FMT from responders to immunotherapy has induced tumor regression in some non-responder CRC patients in case studies). A controlled rat model of T2DM found that FMT significantly improved glucose tolerance, reduced HbA1c, and lowered serum LPS and FFA, accompanied by increased abundance of key SCFA-producing taxa like *Roseburia*, *Akkermansia*, and *Faecalibacterium prausnitzii* [39]. However, in human trials, FMT has yielded inconsistent metabolic outcomes, highlighting the need for further clinical validation [39]. Other innovative approaches include engineered bacteria that deliver anti-cancer payloads to the colon, and *postbiotics* (isolated microbial metabolites) that could mimic the benefits of a healthy microbiome without live organism administration. For example, butyrate-containing postbiotics are being tested for their ability to directly inhibit histone deacetylases in colonocytes, thereby reproducing the anti-proliferative effects of microbial fermentation [84].

**Figure 4:**
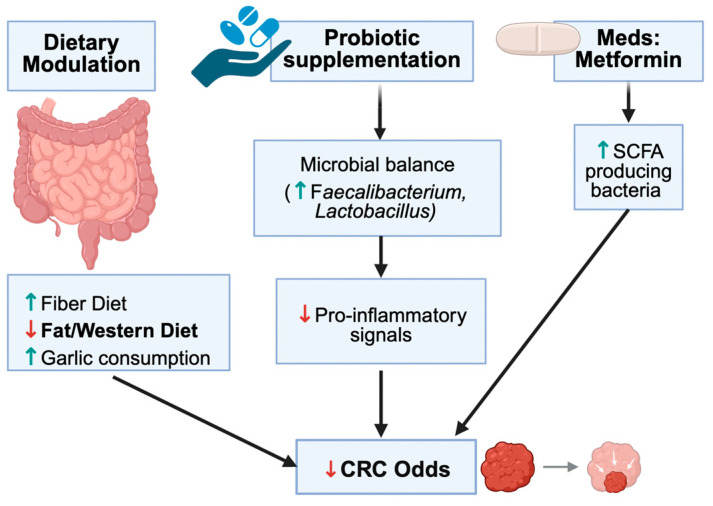
Effects of Dietary and Non-Dietary Interventions on the Gut Microbiota with Relevance to CRC. Summarizes fiber, Mediterranean diets, garlic, probiotics, prebiotics, FMT, and metformin. Interventions generally increase beneficial taxa and SCFAs while reducing pro-inflammatory taxa. Conceptual summary aligned with Table 3. The downward arrows in the schematic, indicate a decrease in the relevant metabolites; conversely, an upward arrow indicates an increase in the relevant metabolite.

**Figure 5:**
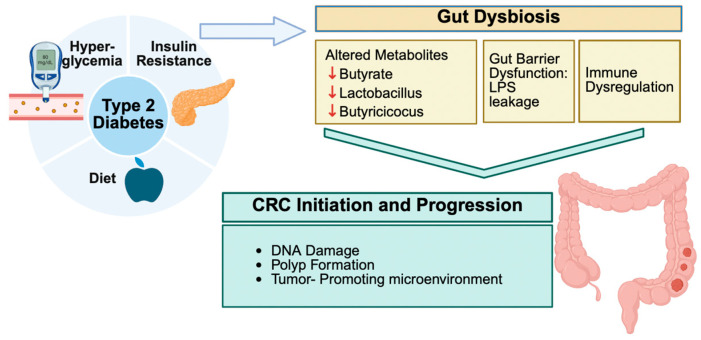
Proposed “common soil” framework integrating metabolic, microbial, and inflammatory pathways in T2DM-CRC interaction. Illustrates the overlap of dysbiosis, metabolic dysfunction, and immune alterations. This framework is hypothesis-generating and synthesizes current evidence; causal pathways require future validation. The downward arrows in the schematic, indicate a decrease in the relevant metabolites.

In summary, targeting the gut microbiota stands out as a compelling two-pronged strategy. By nurturing an eubiotic microbiome, individuals with diabetes can potentially improve metabolic control and concurrently lower the risk of colorectal neoplasia. This has shifted paradigms in preventive medicine—treating the microbiome may be an efficacious form of “immunometabolic therapy” against the dual threats of diabetes and CRC (Figure 4 and Figure 5).

### 4.6. Clinical and Surgical Implications

Our findings carry significant implications for clinical practice, particularly in the fields of gastroenterology and surgery. First and foremost is the potential for earlier CRC screening and risk stratification in patients with T2DM. Given the ~20–30% increased risk of CRC in diabetics and the mechanistic links outlined, clinicians should maintain a higher index of suspicion [7]. Current guidelines do not yet formally differentiate the initiation of screening colonoscopies based on diabetes status. Still, our synthesis suggests that T2DM patients—especially those with long-standing disease or poor glycemic control—constitute a high-risk group who may benefit from earlier or more frequent colonoscopic surveillance. In practice, a patient with T2DM may undergo a colonoscopy at age 45, for example, if additional risk factors are present. Some researchers are even exploring microbiome-based biomarkers to refine risk prediction. Novel analytical tools, such as the Specific Network Information Gain (SNIG) algorithm, can detect subtle shifts in gut microbiota that precede the transition from benign to malignant states [61]. By applying such algorithms to stool samples, it may become feasible to identify “tipping points” in an individual’s microbiome that signal early colorectal carcinogenesis, enabling precision screening. Likewise, improved data imputation methods (e.g., mbImpute) enable more robust microbiome analysis, even with sparse sequencing data, thereby strengthening the reliability of stool diagnostics [43]. In parallel, interventions that actively reshape the microbiota show translational promise. A recent pilot trial demonstrated that FMT improved glycemic control in T2DM patients by enhancing SCFA-producer abundance and lowering systemic endotoxin burden [39]. Such findings raise the possibility that targeted microbiome therapies could eventually reduce both metabolic and oncologic risks. Soon, a combination of clinical factors (such as diabetes status and obesity) with microbiome profiles and perhaps metabolic markers (like serum TMAO or SCFA levels) could form an integrated scoring system to identify patients who require colonoscopy sooner or need preventive interventions.

For surgeons, an appreciation of the gut microbiome’s impact opens up new approaches to perioperative management and long-term outcomes. It is increasingly recognized that the gut microbiota influences surgical recovery; for instance, dysbiosis has been linked to higher rates of postoperative infections, anastomotic leaks, and delayed healing in colorectal surgery [85]. Therefore, modulating the microbiome pre- and post-surgery might improve patient outcomes. Practical steps could include a prehabilitation program with a high-fiber diet or probiotics in the weeks leading up to surgery to optimize the patient’s microbiota. Some surgical centers have initiated small trials administering synbiotics before elective colon resection, observing a reduction in postoperative diarrhea and infection rates [79]. Beyond probiotics, multi-strain formulations are being evaluated in oncology: recent RCTs report that multi-strain probiotics can enhance the efficacy of immune checkpoint inhibitors in solid tumors, potentially through improved antigen presentation and CD8+ T cell priming [55,57]. Such adjunctive strategies could become relevant in CRC patients requiring adjuvant immunotherapy. Additionally, the microbiome’s role in tumor behavior could inform surgical decision-making. For example, tumors enriched with *F. nucleatum* are often right-sided and aggressive; identifying such microbial signatures preoperatively (via stool or biopsy analysis) might prompt more extensive lymph node dissection or vigilant follow-up due to higher recurrence risk. Furthermore, in diabetic CRC patients, controlling blood sugar levels alone may not be sufficient—addressing microbiota health could become part of a holistic management approach. Endocrinologists and surgeons may need to collaborate to ensure that diabetic patients receive dietary counseling or microbiome-focused therapies as an adjunct to standard oncologic care. The implications extend to adjuvant treatment as well. Since certain gut bacteria can inactivate chemotherapeutic drugs or influence the response to immunotherapy, assessing and modulating the microbiome could help personalize treatment. For instance, the presence of *Fusobacterium* in a resected tumor might encourage the use of metronidazole (an antibiotic) alongside chemotherapy to potentially improve outcomes—a strategy currently under investigation. Metabolic agents such as metformin and statins are increasingly viewed in an onco-microbiome context: metformin remodels carcinogenic secondary bile acid pools through microbiota-dependent pathways, while statins may suppress bile acid-transforming microbes, jointly offering metabolic and microbial benefits for CRC prevention [11,45]. In summary, recognizing the gut microbiota as an “invisible organ” influencing surgical patients encourages a more integrative approach: combining metabolic control, nutritional optimization, and microbial management to enhance surgical cure rates and reduce complications in CRC, particularly for those with comorbid T2DM.

### 4.7. Limitations and Knowledge Gaps

Despite the compelling associations detailed in this review, several limitations temper our conclusions and point to areas for caution. First, much of the evidence linking dysbiosis to CRC in diabetics is correlative. Human studies often reveal associations between specific microbes and disease states, but it remains challenging to distinguish cause and effect definitively. While some preclinical experiments (such as fecal transplants that triggered tumor formation in mice) support a plausible causal role, definitive causal links in humans have not yet been established [45]. Encouragingly, some prospective studies are underway—for example, a large Dutch cohort recently revealed that baseline microbiome differences could predict future development of colonic polyps [86]. However, more such studies are needed to confirm that microbiome changes occur before CRC onset in T2DM patients, rather than being consequences of disease or treatment. A related limitation is the difficulty in defining a “healthy” vs. “dysbiotic” microbiome universally. The gut microbiota is highly individualized and influenced by factors such as geography, diet, medication, and genetics. Indeed, microbial composition differences have been noted between Eastern and Western cohorts with T2DM or CRC [87]. One meta-analysis found that after controlling for confounders like diet, stool transit time, and body mass index, some microbes previously thought to be universally associated with CRC (e.g., *Fusobacterium*) were no longer significant, whereas others like *Parvimonas* remained robust markers [8]. This underscores that confounding factors can skew microbiome studies. Many CRC patients receive antibiotics, bowel preps, or metformin (in diabetics), all of which alter the microbiota and could confound study results. We attempted to include studies with careful controls; however, not all literature uniformly adjusts for these variables.

Another limitation lies in the methodologies of microbiome analysis. Different studies employ 16S rRNA sequencing versus shotgun metagenomics, various bioinformatic pipelines, and differing statistical thresholds for “significance” in microbial differences. This heterogeneity can lead to inconsistent results regarding which bacteria are implicated. For instance, some reports highlight an increase in *Lactobacillus* spp. in T2DM, while others find a decrease, likely due to methodological and population differences. The use of advanced methods, such as mbImpute, to handle sparse data is relatively recent [88]. Until such methods are widely adopted, some reported taxonomic changes could be artifacts of data sparsity or sequencing depth issues. Furthermore, most studies focus on bacterial composition; the roles of the fungal (mycobiome) and viral (virome) components are relatively understudied. We noted one report of a higher Basidiomycota/Ascomycota fungal ratio in CRC patients [66]. Still, data on how T2DM might affect the mycobiome (and thereby CRC risk) are scarce. Similarly, bacteriophages and viruses in the gut can influence bacterial populations and gut immunity, representing another layer of complexity that is not captured in our review due to a lack of data.

From a clinical perspective, a limitation is that interventions targeting the microbiome for CRC prevention are still largely experimental. While animal data and small trials are promising, we do not yet have large, randomized controlled trials that definitively prove that altering the microbiota (through diet, probiotics, fecal microbiota transplantation, or FMT) will reduce CRC incidence in individuals with diabetes. Recent RCTs indicate that multi-strain probiotics may enhance immunotherapy responsiveness by improving antigen presentation and CD8+ T cell activity, yet whether such immune modulation translates into CRC prevention in diabetics is unknown [89]. Likewise, early FMT trials improved insulin sensitivity and gut barrier integrity, though long-term benefits remain uncertain [39]. There is also a tension between correlation and causation—for example, metformin users have different microbiomes and lower CRC rates, but is that entirely due to metformin’s microbiome effects or other factors [39]? It is not easy to attribute causality in such scenarios. Our literature review methodology itself could introduce bias, as we included only English-language, published studies. This may lead to publication bias (where negative or inconclusive studies are underreported) and an overrepresentation of particular regions. We also combined evidence from human cohorts and rodent models, which, while complementary, are not directly comparable. Animal models often use extreme diets or genetic strains that may not perfectly mirror the human condition of T2DM or sporadic CRC.

An additional limitation of the current evidence base is that relatively few microbiome studies have systematically accounted for environmental exposures, such as air pollution, particulate matter, and other environmental toxicants, which are now recognized as important determinants of gut microbiota composition [PMID: 37169193]. These exposures can remodel microbial communities, modulate systemic inflammation, and may influence CRC development independently or synergistically with T2DM. Future work should incorporate detailed environmental exposure data when examining microbiota-mediated CRC risk in diabetic populations, to better disentangle host–microbe–environment interactions.

In summary, this review has several important limitations. First, the underlying studies are heterogeneous with respect to design, sample size, microbiome sequencing platforms, bioinformatic pipelines, and adjustment for confounders, precluding formal quantitative synthesis. Second, most human data are cross-sectional, limiting causal inference and making it difficult to disentangle whether dysbiosis precedes, accompanies, or results from T2DM and CRC. Third, several mechanistic links discussed here—particularly those involving specific microbial metabolites, metformin-associated shifts, and immune pathways—are supported primarily by preclinical or small proof-of-concept studies. Finally, key modifiers such as diet, medication use, environmental exposures, and pericolic adiposity are inconsistently measured across cohorts. These limitations underscore the need for large, longitudinal, T2DM-focused cohorts and rigorously designed interventional trials.

### 4.8. Future Research Directions

Building on the current insights, several avenues for future investigation emerge to more fully elucidate and exploit the microbiota’s role in the T2DM–CRC interplay.

**Longitudinal Cohort Studies:** A top priority is establishing prospective cohorts of individuals with T2DM who are tracked for microbiome composition and CRC outcomes over the years. By analyzing serial stool samples, researchers can determine if specific microbial shifts consistently precede the development of colorectal neoplasms. This would move the field from association to prediction, identifying early microbial warning signs of cancer. Efforts like the ongoing NIH Human Microbiome Project’s multi-omics cohort or large biobanks linking microbiome data to cancer registries will be invaluable. Specifically, studies in diverse populations (accounting for diet, ethnicity, geography) are needed, as microbial risk signatures may differ—an aspect highlighted by regional disparities in CRC microbiomes (e.g., distinct microbiota patterns have been noted in Appalachian populations with high CRC rates [8].

**Mechanistic Experiments:** On the laboratory side, more mechanistic research can pin down how exactly dysbiosis drives tumorigenesis. Germ-free animal models colonized with microbiota from diabetic CRC patients (as opposed to healthy controls) could be used to observe differences in tumor initiation under controlled conditions. Such experiments, combined with knockout mice lacking specific immune receptors (e.g., TLR4, IL-6), could clarify which inflammatory pathways are indispensable for microbiota-driven cancer promotion. Additionally, isolating specific microbial metabolites for testing will help, for example, administering TMAO or DCA to rodents to see if they accelerate polyp growth, and conversely testing whether butyrate supplementation can protect against it. The role of microbial genes also deserves attention. Metagenomic analyses might identify particular bacterial virulence or enzyme genes (like those for producing secondary bile acids or genotoxins) that are enriched in diabetic-CRC microbiomes. These genes could become targets for new drugs or probiotics engineered to knock out those functions.

**Microbiota-Based Therapies and Trials:** Microbiota-based therapies represent a particularly promising translational avenue. Early interventional trials of prebiotic and probiotic formulations have demonstrated the feasibility of shifting gut microbiota composition, increasing SCFA production, and modulating inflammatory markers in patients with metabolic syndrome and in individuals undergoing treatment for CRC [PMID: 37929014]. Small-scale studies of faecal microbiota transplantation (FMT) have also suggested that engraftment of donor microbiota can improve insulin sensitivity or enhance responses to cancer therapy in selected settings [PMID: 37929014]. However, these trials remain heterogeneous in design, dose, duration, and endpoints, and very few have specifically targeted patients with T2DM at high risk of CRC. Carefully designed, randomized controlled trials that integrate microbiome, metabolomic, and clinical outcomes in T2DM and CRC populations are required before microbiota-based therapies can be routinely implemented.

**Integration of Multi-omics and AI:** The integration of multi-omics approaches and artificial intelligence (AI) offers a powerful strategy to unravel the complex T2DM–CRC–microbiota nexus. Combining metagenomics, metatranscriptomics, targeted and untargeted metabolomics, and host genomic or epigenomic data can help delineate causal pathways and identify robust, microbiota-derived biomarkers [PMID: 29606345, PMID: 31477928]. Machine-learning algorithms applied to these high-dimensional datasets are already beginning to improve disease classification, predict treatment response, and identify microbially driven metabolic signatures in metabolic and oncologic cohorts [PMID: 26590418, PMID: 31477917]. Extending such AI-driven, multi-omic frameworks to T2DM populations at risk of CRC may enable earlier risk stratification, personalised preventive strategies, and more precise targeting of microbiota-directed interventions.

**Expanding Scope:** Lastly, future studies should broaden the scope beyond the colon. If gut microbiota is affecting systemic inflammation and metabolism, it may well influence other obesity-linked cancers (like liver, pancreatic, or breast cancer). T2DM is a risk factor for several cancers, suggesting that the microbiome may be a unifying thread. Exploring whether similar dysbiosis patterns are found in diabetics who develop other cancers could generalize the concept of microbiome-mediated “field effects” in carcinogenesis. Conversely, we should also examine whether interventions that succeed in CRC could be applied to other metabolic cancers—for example, would a high-fiber diet reduce the risk of hepatocellular carcinoma in NAFLD patients through similar mechanisms, such as an increase in SCFAs and a reduction in inflammation?

In summary, the path forward requires collaborative, interdisciplinary research marrying clinical insight with microbial ecology and molecular biology. As we fill these knowledge gaps, we move closer to a future where we can confidently answer not just whether dysbiosis correlates with CRC risk, but exactly how it causes it and how we can intervene. The ultimate vision is prevention: imagine a world where modifying a person’s gut microbiota from a young age, through diet or safe therapeutics, could significantly reduce their lifetime risk of both diabetes and colorectal cancer. While ambitious, this vision is now within reach if guided by rigorous research.

### 4.9. Conceptual Framework and Conclusions

Synthesizing the evidence, we propose a conceptual framework in which the gut microbiota functions as a central theoretical link bridging metabolic disease and colorectal carcinogenesis. In this model, an unhealthy diet and genetic predispositions precipitate gut dysbiosis, which in turn triggers a cascade of metaflammation—a portmanteau of metabolism and inflammation describing chronic, low-grade inflammatory states common to obesity, T2DM, and cancer [90]. This chronic inflammation (fueled by microbial LPS and impaired gut barrier) sets the stage for genomic instability and cellular proliferation in colonic epithelium, aligning with known hallmarks of cancer. Simultaneously, microbial metabolites act as endocrine and paracrine signals: some (like SCFAs) maintain homeostasis, while others (like DCA and TMAO) serve as tumor promoters. The “Common Soil” hypothesis in oncology posits that disparate diseases can arise from shared environmental roots—here, the gut microbiome is proposed as the fertile soil from which both T2DM and CRC can grow. Our findings support this theoretical framework that interventions improving the soil (the microbiota) can yield benefits in both crops (metabolic and neoplastic outcomes). For instance, enriching SCFA-producing bacteria can improve insulin sensitivity and exert anti-tumor effects, illustrating a single mechanism with dual impact [91]. In essence, the gut microbiota becomes an active player rather than a bystander, not only reflecting the state of its host but actively driving pathophysiological changes across organ systems. Although several studies have evaluated microbial metabolites such as short-chain fatty acids, secondary bile acids, and TMAO in T2DM and in CRC separately, we found relatively few biomarker studies that explicitly stratified CRC-related biomarkers within well-phenotyped T2DM cohorts. Existing data, therefore, point to a plausible convergence of microbial metabolite signatures in T2DM and CRC, but robust, diabetes-stratified biomarker studies—linking microbial products, inflammatory mediators, and clinical CRC endpoints—remain limited and represent a key area for future research.

It is important to emphasize that this framework is dynamic and bidirectional. T2DM can foster an environment (hyperglycemia, altered gut transit, immune changes) that selects for a more pro-carcinogenic microbiota; in turn, that microbiota exacerbates the diabetic state and promotes carcinogenesis, creating a self-reinforcing loop. We also integrate the concept of “the gut microbiome as an endocrine organ”—much like the pancreas or thyroid, the microbiome secretes compounds that travel systemically and influence distant tissues [62]. This concept broadens our theoretical understanding: treatments targeting the microbiota (diet, pre/probiotics) might be seen analogously to hormone therapies, aiming to restore a healthy secretion profile of microbial metabolites (Figure 4 and Figure 5).

## 5. Conclusions

In conclusion, our extensive discussion highlights that gut dysbiosis in T2DM is not a mere epiphenomenon, but rather a causal nexus that significantly elevates colorectal cancer risk. The convergence of compositional changes (loss of beneficial microbes, gain of pathobionts), functional shifts (toxic metabolite accumulation, SCFA depletion), and immune modulation (barrier dysfunction, inflammation, immune evasion) paints a compelling picture of a unified pathophysiology. Importantly, this nexus is actionable. The same microbial characteristics that make the diabetic colon cancer-prone also represent therapeutic touchpoints—leverage points where we can intervene. Dietary fiber, precision probiotics, metabolic drugs like metformin, and novel microbiome therapies can potentially break the chain linking diabetes to colorectal neoplasia. The theoretical framework emerging from current evidence advocates for an interdisciplinary approach: treating the patient’s “microbial organ” alongside their human organs. By doing so, we can move toward truly preventive medicine—mitigating two of the 21st century’s most prevalent diseases with one paradigm shift. As research progresses, we anticipate that managing gut microbiota will become as fundamental in colorectal cancer prevention as managing blood pressure or cholesterol is in cardiovascular prevention. The challenge ahead lies in translating these insights into clinical protocols and public health policies, but the groundwork laid by this and other studies provides a strong impetus. In the coming decade, integration of microbiome health into routine care for diabetics (and indeed, for all patients) could markedly reduce the global burden of colorectal cancer, fulfilling the promise of a more holistic and proactive medical practice.

This review presents compelling evidence that gut dysbiosis in T2DM is not merely a consequence of metabolic disease but a mechanistic driver of colorectal carcinogenesis. Compositional imbalances, metabolic disruptions, and immune dysfunction collectively establish a pro-carcinogenic niche in the colon, characterized by loss of SCFA producers, enrichment of inflammatory taxa, and accumulation of secondary bile acids. These processes are modifiable. Evidence from dietary interventions, pharmacological agents such as metformin, and fecal microbiota transplantation shows that microbiome manipulation can improve metabolic regulation while attenuating tumorigenic risk. Thus, microbiome-directed medicine offers dual potential, enhancing diagnostic precision and shaping new therapeutic strategies at the interface of metabolic and oncologic disease.

Taken together, the available evidence supports the view that gut dysbiosis in T2DM is unlikely to be a mere epiphenomenon. Rather, it appears to represent a mechanistically plausible nexus through which chronic metabolic dysfunction, microbial metabolites, and immune dysregulation converge to influence colorectal carcinogenesis. However, much of the existing literature is cross-sectional and correlational, and key questions regarding causality, temporality, and effect size remain unresolved. Future longitudinal, interventional, and multi-omic studies will be essential to test and refine the “common soil” hypothesis proposed here and to translate microbiota-centred insights into robust preventive and therapeutic strategies for individuals living at the intersection of T2DM and CRC.

## Figures and Tables

**Table 1:** Overview of preclinical and clinical studies examining gut microbiota, metabolic signatures, and colorectal cancer (CRC) in the context of type 2 diabetes mellitus (T2DM) or related metabolic states.

Study	Reference	Model/Context	Key Microbiota Findings	CRC-Related Findings	T2DM/Metabolic Findings	Preventive/Therapeutic Insights
Metagenomic and targeted metabolomic analyses reveal distinct patterns in patients with colorectal cancer and type 2 diabetes mellitus	[18]	Human patients; DCRC vs. CRC vs. controls	DCRC group shows enrichment of *Peptostreptococcus*, *Porphyromonas*, *Parvimonas*, *Eggerthella*, *Hungatella*, *Veillonella*; depletion of *Butyricicoccus*, *Lactobacillus spp.*, *Paraprevotella* and other butyrate producers	Compounded dysbiosis and altered bile acid/SCFA profiles may favor colorectal carcinogenesis	DCRC patients show diabetes-related metabolic dysregulation superimposed on CRC, with altered BA–SCFA interactions	Highlights targeting BA–SCFA pathways and dysbiotic taxa in DCRC as a potential preventive/therapeutic strategy
Garlic consumption in relation to colorectal cancer risk and to alterations of blood bacterial DNA	[19]	Human; observational	Higher garlic intake associated with distinct blood bacterial DNA profiles suggestive of systemic microbiome modulation	Medium/high garlic intake significantly associated with lower CRC risk, particularly rectal cancer	Garlic intake associated with more favorable inflammatory/metabolic profile	Supports garlic-rich diets as chemopreventive via microbiota and immune modulation
Short-chain fatty acids reprogram metabolic profiles with the suppression of colorectal cancer cell species production in human colorectal adenocarcinoma cells	[20]	In vitro; CRC cell lines	SCFAs (especially butyrate) act as key microbial metabolites influencing cancer cell metabolism	Butyrate and other SCFAs suppress CRC progression by reprogramming glycolysis and mitochondrial metabolism, increasing ROS and inducing apoptosis	SCFAs are central to energy homeostasis and may improve insulin sensitivity (relevant to T2DM)	Supports strategies to enhance SCFA production via diet/microbiota modulation as CRC-preventive
The Association Between Prebiotic Fiber Supplement Use and Colorectal Cancer Risk and Mortality in the Women’s Health Initiative	[21]	Human; prospective cohort	Prebiotic fiber expected to enrich *Bifidobacterium* and other SCFA producers (microbiota not directly sequenced in all)	Prebiotic fiber supplement use associated with modestly lower CRC risk and improved survival after CRC	Fiber intake linked to better metabolic profile, indirectly relevant to T2DM	Encourages high-fiber/prebiotic intake as adjunct in CRC prevention and metabolic health
Altered intestinal microbiota associated with colorectal cancer	[22]	Human; CRC vs. controls	CRC patients have decreased SCFA-producing taxa (*Faecalibacterium*, *Roseburia*) and increased potentially pathogenic bacteria	Defines characteristic CRC-associated dysbiosis	Overlaps with patterns described in metabolic disorders but diabetes not primary focus	Supports microbiota-targeted prevention (diet, probiotics) aimed at restoring beneficial taxa
Relationship between obesity-related colorectal tumors and the intestinal microbiome: an animal-based trial	[23]	Mouse; obesity-related CRC model	Obesity-related CRC tumors show distinct microbial signatures vs. lean CRC, with enrichment of obesogenic and pro-inflammatory taxa	Obesity-associated dysbiosis correlates with greater tumor burden and more aggressive CRC	Obesity/metabolic dysfunction (T2DM-like) interact with dysbiosis to promote CRC	Suggests weight loss and microbiota modulation to reduce obesity-related CRC risk
Fibre-rich Foods to Treat Obesity and Prevent Colon Cancer trial in obese patients with a history of noncancerous adenomatous polyps	[24]	Human; interventional, high-fiber diet	Fiber-rich diet increases SCFA-producing taxa and reduces pro-inflammatory/pathogenic bacteria	Reduces markers associated with colon cancer risk (e.g., polyp recurrence, inflammatory markers)	Improves weight, insulin sensitivity, and lipids in obese participants	High-fiber diets offer dual benefit: colon cancer prevention and metabolic improvement
Impact of a high-fat diet on intestinal stem cells and epithelial barrier function in middle-aged female mice	[25]	Mouse; high-fat diet (HFD)	HFD reduces microbial diversity, enriches pro-inflammatory taxa, and impairs gut barrier integrity	Barrier dysfunction and inflammation under HFD create a milieu favorable for CRC	HFD promotes insulin resistance and T2DM-like metabolic dysfunction	Suggests limiting HFD and increasing fiber to preserve barrier function and reduce CRC/T2DM risk
Impacts of pre-existing diabetes mellitus on colorectal cancer in a mice model	[26]	Mouse; T2DM + CRC	Diabetic CRC mice exhibit distinct dysbiosis vs. non-diabetic CRC, with shifts in SCFA producers and pathobionts	Pre-existing diabetes exacerbates tumor growth, aggressiveness, and inflammatory signaling	Shows that diabetes-induced metabolic changes and dysbiosis synergistically worsen CRC outcomes	Highlights need for aggressive metabolic control and possible microbiota modulation in diabetic CRC
Impacts of pre-existing diabetes mellitus on colorectal cancer in a mice model	[26]	Human; T2DM + CRC	Enrichment of bile acid-transforming bacteria and depletion of beneficial taxa	BA dysregulation (↑ secondary bile acids like DCA) associated with higher CRC risk and aggressiveness	T2DM-related metabolic alterations intersect with BA–microbiota axis	Suggests BA-targeted therapies and microbiota modulation as preventive/adjuvant approaches in T2DM-associated CRC
Impacts of pre-existing diabetes mellitus on colorectal cancer in a mice model	[26]	Mouse; HFD + tumor/cachexia	HFD and tumor burden reshape microbiota; specific taxa correlate with cachexia and systemic inflammation	Microbiota-mediated effects aggravate tumor progression and cachexia	Links diet-induced dysbiosis, liver dysfunction, and metabolic imbalance	Indicates diet/microbiota interventions could mitigate cachexia and improve cancer outcomes
Adipose tissue inflammation by recruitment of distinct neutrophils and its resolution by Akkermansia muciniphila and its extracellular vesicles	[27]	Mouse; microbiota-directed	Obesity reduces *Akkermansia muciniphila*; supplementation with *A. muciniphila* or its EVs reshapes microbiota and improves barrier	Not directly CRC-focused, but reduces inflammatory milieu relevant to carcinogenesis	Ameliorates obesity-related inflammation and metabolic impairment	Provides rationale for *A. muciniphila*/EV-based therapies to restore gut barrier, reduce inflammation, and indirectly lower CRC risk
Akkermansia muciniphila: A new hope in obesity prevention and treatment…	[28]	Human/animal; review	Summarizes evidence linking *A. muciniphila* to healthy mucus layer, gut barrier, and microbial balance	Suggests possible protective role against carcinogenesis via barrier and immune modulation	Strongly linked to improved metabolic outcomes (obesity, insulin sensitivity)	Proposes *A. muciniphila* as a candidate probiotic/next-gen therapy for obesity, T2DM, and possibly CRC risk reduction
Metformin-induced reductions in tumor growth involves modulation of the gut microbiome	[29]	Mouse; metformin + tumor model	Metformin reshapes the gut microbiota, increasing beneficial taxa and altering SCFA/BA profiles	Metformin reduces tumor growth, partly through microbiota-dependent mechanisms	Confirms metformin’s glucose-lowering and insulin-sensitizing effects with added microbiota-related actions	Supports repurposing metformin as adjuvant therapy in metabolically dysregulated cancer patients
Dietary Factors: Major Regulators of the Gut’s Microbiota	[30]	Human/animal; review	Reviews how macronutrients, fiber, and specific foods regulate gut microbiota	Summarizes diet–microbiota–CRC links	Links diet-induced dysbiosis to obesity, insulin resistance, and T2DM	Advocates high-fiber, plant-rich diets and limited processed meat to promote eubiosis and reduce CRC/metabolic risk
Comparative study of effect of Akkermansia muciniphila and its extracellular vesicles on Toll-like receptors and tight junction	[31]	In vitro/preclinical; microbiota-directed	*A. muciniphila* and its EVs improve microbial balance and increase tight junction protein expression	Not directly CRC-focused, but improved barrier and reduced inflammation are protective against carcinogenesis	Relevant to T2DM via reduced endotoxemia and inflammatory burden	Suggests *A. muciniphila*/EV-based approaches to restore gut barrier and immune–microbiota homeostasis

↑ = increase in.

## Abbreviations

The following abbreviations are used in this manuscript:

CRC	Colorectal Cancer
T2DM	Type 2 Diabetes Mellitus
SCFA	Short Chain Fatty Acids
DCA	Deoxycholic Acid
TMAO	Trimethylamine-N-oxide
FMT	Fecal Microbiota Transplantation
EOCRC	Early-Onset Colorectal Cancer
LPS	Lipopolysaccharide
NF-κB	Nuclear Factor Kappa B
IL	Interleukine
TNF-ɑ	Tumor Necrosis Factor-alpha
GLP-1	Glucagon-Like Peptide 1
IGF-1	Insulin-Like Growth Factor 1
